# Physical activity, sedentary time and sleep duration: associations with body composition in 10–12-year-old Estonian schoolchildren

**DOI:** 10.1186/s12889-018-5406-9

**Published:** 2018-04-13

**Authors:** Eva-Maria Riso, Merike Kull, Kerli Mooses, Jaak Jürimäe

**Affiliations:** Institute of Sports Sciences and Physiotherapy, University of Tartu, 5 Jakobi St, 51014 Tartu, Estonia

**Keywords:** Moderate-to-vigorous physical activity, Duration of sedentary time and sleep, Body composition

## Abstract

**Background:**

Physical activity, sedentary time, and sleep duration have been associated with body composition among children. The purpose of the present study was to assess the associations of objectively determined daily physical activity, sedentary time, sleep duration and body composition indices in 10–12-year-old children.

**Methods:**

Two hundred and eleven schoolchildren (96 boys and 115 girls) aged 10.9 ± 0.7 years participated in this study. Objective physical activity intensity and sedentary levels were measured for seven days by accelerometry. Sleep duration was self-reported. Percentage of body fat, waist-to-height ratio and fat free mass were calculated from measured anthropometric parameters. Multiple linear regression models were used to examine the associations between sleep duration, moderate-to-vigorous physical activity (MVPA), vigorous physical activity (VPA) level and body composition indices.

**Results:**

Boys exceeded girls (*p* < 0.05) in time spent in MVPA and VPA levels. Only 4.3% of the children met the current daily recommendation of at least 60 min MVPA per day. Sleep duration, MVPA and VPA had a negative association with percentage of body fat and waist-to-height ratio. Vigorous physical activity had a positive association with fat-free mass. Sedentary time had a positive association with percentage of body fat and negative association with fat-free mass.

**Conclusions:**

The present study suggests that both sleep duration and MVPA are associated with body composition parameters. Higher levels of MVPA are associated with lower percentage of body fat and waist-to-height ratio regardless of sleep duration. Sedentary time is associated with higher values of percentage of body fat and lower fat-free mass independently of sleep duration.

## Background

Insufficient levels of moderate-to-vigorous intensity physical activity (MVPA), high levels of sedentary time, and short sleep duration have all been associated with higher levels of adiposity among children and adolescents in several recent investigations [1–3]. In contemporary society the sleep duration of children is often insufficient and sleep debt occures, which may be connected with sleep-mediated outcomes such as being overweight or obese [4, 5]. Previous studies in both preschool and primary school children have shown that sufficient sleep duration is positively associated with the level of physical activity and associates with lower risk to be overweight [6, 7]. In a very few studies, PA, sedentary time and sleep duration have been investigated at the same time among 9–11-year-old children [8, 9]. However, the associations among PA, sedentary time and sleep duration with excess adiposity in 10–12-year-old children should be more precisely defined as the results of previous studies are rather controversial [8, 9]. For example, Chaput et al. [8] found that higher levels of MVPA are associated with lower adiposity regardless of sedentary time and sleep duration in 9–11-year-old children. In contrast, Hjorth et al. [9] reported that low PA and short sleep duration were independently associated with higher fat mass index in 8–11-year-old children. As sleep duration influences physical well-being in adolescents [4] it is necessary to evaluate the associations of sleep duration and PA with body composition parameters. In the light of abovementioned findings it could be expected that both sleep duration and PA might be associated with healthy body composition.

Early puberty is a formation period of healthy lifestyle habits and thus it is important to explore the associations between movement behaviours and sleep duration with adiposity values [9]. Furthermore, puberty has been reported to be the period when PA decreases dramatically in boys and girls [10, 11]. It appears also that maturation level is associated with a changed sleep pattern [12] in addition to changes in PA level [10]. Accordingly, better knowledge of combined associations between these habits may be useful to create intervention programs to prevent being overweight and increase PA level in children entering puberty. The purpose of the present study was to assess the amount of daily PA at different intensity levels, sedentary time, self-reported sleep duration and body composition in a specific sample of children entering puberty; and to determine the relationships of objectively measured PA, sedentary time and sleep duration with body composition indices in 10–12-year-old children. Possible differences in PA, sedentary time and sleep duration between boys and girls together with differences in adiposity were also examined.

## Methods

### Participants

The study sample consisted of 13 randomly chosen Estonian schools all over the republic. All children from the second school level (mean age 10.9 ± 0.7 years) of selected schools and their parents received written information about the study. Four hundred and thirty-two children agreed to participate. Three hundred and fifty-three children were randomly chosen from consented students for measuring objective PA with accelerometer and to obtain anthropometrical data. From those children, 142 students did not provide valid accelerometer data and were excluded from the data analysis. Those children did not differ from those entered into the analysis in terms of age, gender and body mass index (BMI) (*p* > 0.05) [13]. In total, valid accelerometer data and anthropometrical measurements were obtained from 211 children (96 boys and 115 girls). Written informed consents from parent and child were obtained from all participants. The study was approved by the Medical Ethics Committee of the University of Tartu, Tartu, Estonia, approval no 2-42 T 7.

### Anthropometric measurements

All measurements were carried out in the school settings. Body mass and height were measured using medical digital scales (A&D Instruments, Abington, UK) and portable stadiometer (Seca 213, Hamburg, Germany) to the closest 0.05 kg and 0.1 cm, respectively, with the subject wearing light clothing without shoes. BMI was calculated as body mass (kg) divided by body height squared (m^2^). Age-adjusted BMI cut-off points were used to define overweight and obese subjects [14]. The boys were considered to be overweight when their BMI in 10, 11 and 12 years was accordingly 19.84, 20.55 and 21.22 kg/m^2^ and the overweight threshold for the girls of the same age was 19.86, 20.74 and 21.68 kg/m^2^ [14]. Anthropometric parameters were measured according to the protocol recommended by the International Society for the Advancement of Kinanthropometry [15]. Four skinfold thicknesses (*triceps, biceps, subscapular, supra-iliac*) were measured in triplicate on the right side of the body with a Holtain caliper (Crymmych, UK) to the nearest 0.2 mm using standard procedures [15]. Prior to the measurements in school settings, the members of data collecting team trained the measurements procedures as shown in ISAK protocol [15] until for all the skinfold thickness measurements, intra-observer technical errors were smaller than 1 mm and reliability greater than 95%. Interobserver reliability for skinfolds was higher than 90% [13]. In every school, the same trained investigators made all skinfold thickness measurements. All measured skinfolds were also summarized (sum of skinfolds) as an indicator of total subcutaneous body fat [16]. The percentage of body fat (body fat %) was calculated from *triceps* and *subscapular* skinfold thicknesses using the Slaughter et al. [17] equations. Fat mass was calculated using value of body fat %. In addition, fat free mass (FFM) was calculated [18]. Waist circumference was measured using a metal tape from the Centurion kit (Rosscraft, Canada) [15] and waist-to-height ratio (WHtR) was calculated as an indicator of central adiposity [18]. This index is important to identify children with high cardiometabolic risk, and a WHtR cut-point ≥0.5 is associated with increased cardiometabolic risk [8]. The used methods are previously described elsewhere [13].

### Physical activity measurements

The Actigraph GT3X accelerometer (ActiGraph LLC, Pensacola, FL, USA) was used to objectively monitor PA and sedentary time during waking hours. Children wore the device on a belt around the waist for 7 consecutive days. Children were asked to remove the device for aquatic activities. Study staff instructed children on how to wear the device. A valid recording for PA and sedentary time required at least 3 days (including at least one weekend day) of at least 10 h of wake/wear time per day [19]. The accelerometer data were analysed using the activity counts of 15 s epochs. For the analyses of accelerometer data, all night activity and all sequences of 20 min or more of consecutive zero counts were excluded from each individual’s recording [19]. Time spent in sedentary behaviour was characterized by < 100 counts per minute [18–20]. The time spent in moderate PA (MPA) and vigorous PA (VPA) was calculated based upon the Evenson et al. [20] cut-offs of 2000 and 4000 counts per minute, respectively. Each individual’s accumulated PA was categorized into different intensities, and average minutes of sedentary time, MPA and VPA over valid days were calculated subsequently. Time spent in MVPA was calculated as the sum of MPA and VPA. The daily percentage of all PA intensity levels was calculated after summarizing the time spent in every intensity level including sedentary time [21]. In order to meet current PA guidelines, 60 min of MVPA was required for every single day of PA assessment [21, 22]. In addition, the number of children was calculated whose average MVPA level over all measured days was 60 min or more [19]. Similar registration of PA activity was previously used in recent study of Riso et al. [13].

### Sleep duration

The parents and children were instructed to keep a diary for bedtime during the whole week in which the accelerometer was worn [9]. Sleep duration was only considered valid if it was measured for a minimum of 3 weekdays and 1 weekend day [9].

### Maturity assessment

Biological age was calculated according to gender-specific equations developed by Mirwald et al. [23] which include body height, body mass, leg length, sitting height, chronological age, and their interaction, to estimate the maturity offset or number of years away from peak height velocity (PHV). The maturity offset was used to estimate the age at estimated PHV (estimated age at PHV = chronological age-maturity offset). The average maturing age was obtained using mean estimated age at PHV (APHV) of the sample ± standard deviation [SD] [24].

### Statistical analysis

Data analysis was made using the SPSS version 20.0 for Windows (SPSS, Inc., Chicago, IL, USA). Descriptive statistics are presented as mean (±SD). All variables were checked for normality before the analysis using Kolmogorov-Smirnoff method. Group differences between means were analysed with Mann-Whitney U test. Effect size (ES) was calculated and was considered to be small if ES > 0.1, moderate if ES > 0.3 or large if ES > 0.5. The variance inflation factors between variables were < 5, suggesting that multicollinearity was not a problem in the models. Multiple linear regression models were used to examine the independent associations of sleep duration, PA of different intensities (time spent sedentary or in MVPA and VPA) and sleep duration with body composition indices (body fat%, WHtR, FFM) [8].

The primary model (Model 1) was unadjusted. Model 2 was adjusted for age and gender [25]. Model 3 consisted of Model 2 plus APHV as an indicator of maturity status [26]. In Model 4, the mean value of sleep duration during the week was added to the Model 3. The significance level was set at *p* < 0.05.

## Results

There were no differences in age, height, body mass, BMI and APHV between boys and girls studied (Table 1). Waist circumference (ES = 0.15), WHtR (ES = 0.16) and FFM (ES = 0.22) were significantly lower in girls compared with boys (*p* < 0.05). The differences in waist circumference (ES = 0.15), WHtR (ES = 0.16) and FFM (ES = 0.22) were small in magnitude, but significant (*p* < 0.05) in girls compared with boys, while girls had moderately higher sum of skinfolds (ES = 0.44) and body fat% (ES = 0.36) values in comparison with boys (*p* < 0.05). Twenty-six percent (*n* = 55) of children were classified as overweight or obese based on the international cut-off points including 39 overweight (18.5% of the whole sample) and 16 obese (7.5% of the whole sample) children [14]. Overall, 13.7% (*n* = 29) of children studied had a WHtR of ≥0.5.

**Table 1:** Descriptive data of study sample, boys and girls

	Boys (*n* = 96)	Girls (*n* = 115)	All (*n* = 211)
Age (yrs)	11.07 ± 0.74	10.86 ± 0.68	10.96 ± 0.72
Body weight (kg)	45.4 ± 12.52	43.5 ± 11.4	44.35 ± 11.9
Height (cm)	151.3 ± 8.84	149.9 ± 8.9	150.6 ± 8.9
BMI	19.56 ± 3.72	19.14 ± 3.78	19.33 ± 3.75
Waist circumference (cm)	66.45 ± 9.35	64.14 ± 8.89*	65.19 ± 9.16
Waist-to-height ratio	0.44 ± 0.05	0.43 ± 0.05*	0.43 ± 0.05
Fat free mass (kg)	36.1 ± 6.75	33.45 ± 7.39*	34.6 ± 7.21
Body fat percent	18.2 ± 8.65	22.17 ± 5.51*	20.37 ± 7.37
Sum of skinfolds	40.4 ± 26.1	59.7 ± 29.2*	51.0 ± 29.4
APHV	13.78 ± 0.51	13.72 ± 0.42	13.75 ± 0.461

Value are presented as mean ± SD; * - *p* < 0.05 as compared with boys

Average wearing time of the accelerometer was 794 ± 53 min/day (Table 2). About 4.3% of the children met the current daily MVPA recommendations of 60 min or more MVPA per day [22]. At the same time, 36.5% of children obtained 60 min or more MVPA minutes when the average MVPA over measured days is evaluated. Boys exceeded (*p* < 0.05) girls in time spent in MVPA (ES = 0.17) and VPA (ES = 0.18) levels (Table 2). The average sleep duration of the whole week was approximately 9.5 h per day. Most of the accelerometer wearing time was spent being sedentary (62%) and only 6.9% of the total time accounted for MVPA in which more time was spent in MPA (4.7%).

**Table 2:** Sleep duration, sedentary time and different PA intensity levels of study participants

	Boys (*n* = 96)	Girls (*n* = 115)	Total sample (*n* = 211)
Boys (*n* = 96)			
Sleep duration (min)	550 ± 41	559 ± 41	555 ± 41
Sedentary time (min)	490 ± 55	498 ± 55	494 ± 55
Vigorous PA (min)	20 ± 13#	16 ± 9	18 ± 11
MVPA (min)	60 ± 24#	51 ± 20	55 ± 22
Total PA (min)	302 ± 61	299 ± 54	300 ± 57
Average measured time (min)	792 ± 58	796 ± 49	794 ± 53

Values are presented as mean ± SD; # - *p* < 0.05 as compared with girls

Associations of sleep duration and different PA levels with body composition values are demonstrated in Table 3. The average weekly sleep duration was not associated (*p* > 0.05) with body composition parameters in unadjusted models (Table 3). The average sleep duration was negatively associated with body fat% and FFM after adjusting for age and gender (Model 2). These associations remained significant after further adjustment for APHV (Model 3). In unadjusted model, sedentary time was not associated with body composition parameters, while MVPA and VPA had a negative association with body fat% and WHtR. After adjustment for age and gender, sedentary time had a positive association with body fat% and negative association with FFM. Both MVPA and VPA had a negative association with body fat% and WHtR (Model 2). The addition of another covariate, APHV, did not change the associations between MVPA and VPA with body fat%. Further adjustment for average sleep duration did not change the associations between sedentary time and MVPA with body composition parameters (Model 4).

**Table 3:** Associations between sleep duration, sedentary time and different PA intensities with body composition parameters

		Body fat%			WHtR			FFM	
	R^2^	β	P	R^2^	β	P	R^2^	β	P
Sleep duration									
*Model 1*	0.001	−0.024	0.726	0.005	− 0.069	0.321	0.003	−0.055	0.441
*Model 2*	0.111	−0.050	0.000	0.016	−0.059	0.327	0.143	−0.028	0.000
*Model 3*	0.139	−0.052	0.000	0.042	−0.058	0.067	0.327	0.023	0.000
Sedentary time									
*Model 1*	0.003	0.059	0.393	0.005	−0.071	0.300	0.007	−0.084	0.225
*Model 2*	0.111	0.037	0.000	0.019	−0.063	0.258	0.146	−0.072	0.000
*Model 3*	0.137	0.022	0.000	0.038	−0.053	0.093	0.332	−0.110	0.000
*Model 4*	0.139	0.010	0.000	0.047	−0.078	0.077	0.341	−0.124	0.000
MVPA									
*Model 1*	0.062	−0.248	0.000	0.023	−0.153	0.026	0.000	−0.020	0.771
*Model 2*	0.144	−0.189	0.000	0.045	−0.178	0.021	0.142	−0.032	0.000
*Model 3*	0.168	−0.181	0.000	0.067	−0.183	0.006	0.320	−0.014	0.000
*Model 4*	0.174	−0.196	0.000	0.077	−0.194	0.006	0.327	−0.021	0.000
VPA									
*Model 1*	0.100	−0.316	0.000	0.044	−0.209	0.002	0.001	0.022	0.745
*Model 2*	0.174	−0.260	0.000	0.071	−0.242	0.002	0.141	0.000	0.000
*Model 3*	0.200	−0.259	0.000	0.090	−0.241	0.001	0.320	−0.002	0.000
*Model 4*	0.209	−0.276	0.000	0.101	−0.253	0.001	0.327	−0.007	0.000

Model 1 – unadjusted; Model 2 – adjusted for age and gender; Model 3 – adjusted for age, gender and maturity; Model 4 – adjusted for age, gender, maturity and sleep duration

## Discussion

Associations between time spent in different PA levels, sleep duration and body composition indicators in 10–12-year-old children have still been little studied. Therefore, it is necessary to examine the associations objectively measured PA levels and sleep duration on the body fatness ([1–3] as the lack of PA, shortened sleep duration and childhood obesity are serious health problems during growth and maturation [8, 9]. The main findings of our study were that higher MVPA and consequently VPA levels were associated with lower body fat% and WHtR values in 10–12-years-old children regardless of the sleep duration (see Table 3). The associations between PA levels and body composition parameters were stronger in our study as compared with associations among sedentary time, showing that PA has more effect on healthy body composition in our study sample. The importance of PA in daily schedule of schoolchildren must be emphasized based on our results. Although only 4.3% of the schoolchildren were compliant with current daily PA recommendations, 36.5% of children were physically active for more than 60 min according to the average MVPA over the observation period. At the same time, children still spent over half of their day being sedentary (62%). These results emphasize the decisive role of MVPA in influencing adiposity status in children and support the suggestion that the future efforts for obesity prevention should focus on increasing daily MVPA and reducing daily sedentary time in children entering puberty [8].

In comparison with our results, Cooper et al. [27] described that in all 20 studied countries only 9% boys and 1.9% 5–17-year-old girls met the guidelines of achieving 60 min of MVPA every day. Moreover, the comparison of different PA studies among children and youth is problematic because different methods to assess PA intensity are often used [27]. Using different cut-off points to distinguish different PA levels precludes objective comparison of results across different studies. Too many sedentary activities among young children is a growing problem all over the world and thus there is a need to examine the sedentary behaviour habits in children. Sedentary time of schoolchildren has been assessed in children with different age groups [8, 28]. Hjorth et al. [9] found that 10-year-old Danish children spent approximately 52.4% of their day being sedentary, whereas Spittaels et al. [28] registered also that about 52% of the day was engaged with sedentary activities in 8–13-year-old Belgian children. As compared with above-mentioned results, the sedentary time of our studied 10–12-year-old Estonian schoolchildren (62%) is higher as compared to their peers in other countries. It is previously found that girls are more sedentary than boys [11]. In our study, the duration of sedentary time was not different between 10 and 12-year-old boys and girls. The results of our study as well as study of Spittaels et al. [28] show that it is important to reduce sedentary time by replacing a part of sedentary time with more intensive physical activities among growing children.

Significant differences in MVPA and VPA levels were found between boys and girls while the boys exceeded girls in time spent on MVPA and VPA level (Table 2). This finding is in accordance with several studies which have revealed that boys are more physically active than girls, especially on MVPA and VPA levels already at the primary school age [8, 19]. The participants of our study spent on the average 18 min per day in VPA. It has been suggested that a minimum of 15 min per day of VPA is desired to reduce the risk of developing overweight or obesity in later puberty [24].

To date, relatively few studies have examined the associations of sleep duration and PA levels with body composition indicators among schoolchildren [8, 9] and the age of participants of previous studies differed from our sample. The specific sample of 10–12-year-old children entering puberty was examined in study and we found that overall sleep duration was negatively associated with body fat% and positively associated with FFM after adjustment for several confounders (see Table 3). This is in accordance with previous findings, which have shown negative associations between overall sleep duration and body fat% [8]. In our study, the average sleep duration showed negative associations with MVPA, VPA and sedentary time, which is controversial with previous studies [6, 7]. Thus we cannot state that longer sleep duration had been ensured more physical activity among participants of our study. Recent investigations have shown that WHtR, which is an indicator of central adiposity, is a useful index to identify children with high cardiometabolic risk [18]. In current study, the average sleep duration was not associated with WHtR even after controlling for several confounders (Table 3). These results are in contrast with previous investigations showing negative association between sleep duration and WHtR [8].

Sedentary time had a positive association with body fat% after adjustment for all confounders (see Table 3), similarly with previous studies [8, 13]. In their study with 8–11-year-old children, Hjorth et al. [9] found that sedentary time was positively associated with fat mass index, which was used as an indicator of body fatness. Above-mentioned findings suggest that decreasing sedentary time is important for achieving a healthy body composition during growth in children entering puberty. MVPA and VPA had negative associations with body fat% and WHtR (see Table 3). Similar associations have recently been found by Riso et al. [13] with 7–9-year-old children and Chaput et al. [8] with 9–11-year-old children.

It is shown previously by several authors that children who spent more time on VPA level gain plausibly less weight over time and that VPA is considered to be most strongly negatively associated with different indices of body fatness [24, 25]. The findings of our study were in accordance with findings of Steele et al. [25] indicating that time spent in MVPA and VPA level was negatively associated with indices of adiposity. Comparably to our results, a positive association was found between VPA and FFM after adjusting for FM, age and gender in the study with 14–15-year-old adolescents [26]. Our recent study [13] with 7–9-years old children demonstrated similar directions which could be explained by the standpoint that the longer duration of MVPA has a greater effect on muscular component of child’s body [29]. PA could decrease FM by increasing total energy expenditure and PA at higher intensity levels can influence positively the muscle mass [26]. At the same time the total energy use intensifies due to the metabolic needs of increased muscle mass [30].

The present study has some limitations. Sleep duration was self-reported while any self-report is a subject of bias and sleep disturbances were not registered. The cross-sectional design of the study does not allow to reflect causal relationships of the observed associations. The body composition was measured indirectly by anthropometric method using skinfolds [17]. However, the strengths of our study are a sample selected from 13 schools from different counties of Estonia, the use of accelerometers to objectively measure PA, and the use of body composition and objectively measured PA values as confounders when analysing associations between PA, sleep duration and body composition variables.

## Conclusions

The present study demonstrates that both sleep duration and MVPA are independently associated with body composition parameters. Higher levels of MVPA are associated with lower body fat% and WHtR regardless of sleep duration. Sedentary time is associated with higher values of body fat% and lower FFM independently of sleep duration.

## Abbreviations

% body fat: Percentage of body fat
BMI: Body mass index
FFM: Fat free mass
FM: Fat mass
LPA: Light physical activity
MPA: Moderate physical activity
MVPA: Moderate-to-vigorous physical activity
NW: Normal-weight
OW: Overweight
PA: Physical activity
SD: Standard deviation
VPA: Vigorous physical activity
WHtR: Waist-to-height ratio
